# Progress and challenges of integrated drug efficacy surveillance for uncomplicated malaria in Thailand

**DOI:** 10.1186/s12936-021-03791-2

**Published:** 2021-06-09

**Authors:** Prayuth Sudathip, Aungkana Saejeng, Nardlada Khantikul, Thannikar Thongrad, Suravadee Kitchakarn, Rungniran Sugaram, Cheewanan Lertpiriyasuwat, Darin Areechokchai, Deyer Gopinath, David Sintasath, Pascal Ringwald, Sathapana Naowarat, Niparueradee Pinyajeerapat, Maria Dorina Bustos, Jui A. Shah

**Affiliations:** 1 Division of Vector Borne Diseases, Department of Disease Control, Ministry of Public Health, Nonthaburi, Thailand; 2 Office of Disease Prevention and Control Region 1, Chiang Mai, Thailand; 3 World Health Organization, Nonthaburi, Thailand; 4 U.S. President’s Malaria Initiative, Regional Development Mission for Asia, United States Agency for International Development, Bangkok, Thailand; 5 World Health Organization, Geneva, Switzerland; 6 Inform Asia: USAID’s Health Research Program, RTI International, Bangkok, Thailand

**Keywords:** Malaria elimination, Surveillance, Drug efficacy, Antimalarial, Drug resistance

## Abstract

**Background:**

Integrated drug efficacy surveillance (iDES) was formally introduced nationally across Thailand in fiscal year 2018 (FY2018), building on a history of drug efficacy monitoring and interventions. According to the National Malaria Elimination Strategy for Thailand 2017–2026, diagnosis is microscopically confirmed, treatment is prescribed, and patients are followed up four times to ensure cure.

**Methods:**

Routine patient data were extracted from the malaria information system for FY2018–FY2020. Treatment failure of first-line therapy was defined as confirmed parasite reappearance within 42 days for *Plasmodium falciparum* and 28 days for *Plasmodium vivax*. The primary outcome was the crude drug efficacy rate, estimated using Kaplan–Meier methods, at day 42 for *P. falciparum* treated with dihydroartemisinin–piperaquine plus primaquine, and day 28 for *P. vivax* treated with chloroquine plus primaquine; day 60 and day 90 efficacy were secondary outcomes for *P. vivax*.

**Results:**

The proportion of patients with outcomes recorded at day 42 for *P. falciparum* malaria and at day 28 for *P. vivax* malaria has been increasing, with FY2020 follow-up rates of 61.5% and 57.2%, respectively. For *P. falciparum* malaria, day 42 efficacy in FY2018 was 92.4% (n = 249), in FY2019 93.3% (n = 379), and in FY2020 98.0% (n = 167). *Plasmodium falciparum* recurrences occurred disproportionally in Sisaket Province, with day 42 efficacy rates of 75.9% in FY2018 (n = 59) and 49.4% in FY2019 (n = 49), leading to an update in first-line therapy to pyronaridine–artesunate at the provincial level, rolled out in FY2020. For *P. vivax* malaria, day 28 efficacy (chloroquine efficacy) was 98.5% in FY2018 (n = 2048), 99.1% in FY2019 (n = 2206), and 99.9% in FY2020 (n = 2448), and day 90 efficacy (primaquine efficacy) was 94.8%, 96.3%, and 97.1%, respectively.

**Conclusions:**

In Thailand, iDES provided operationally relevant data on drug efficacy, enabling the rapid amendment of treatment guidelines to improve patient outcomes and reduce the potential for the spread of drug-resistant parasites. A strong case-based surveillance system, integration with other health system processes, supporting biomarker collection and molecular analyses, and cross-border collaboration may maximize the potential of iDES in countries moving towards elimination.

## Background

The Greater Mekong Subregion (GMS) is the global epicentre of anti-malarial drug resistance. High failure rates for artemisinin-based combination therapy (ACT) against *Plasmodium falciparum* have been reported throughout the region, associated with resistance to both artemisinins and their partner drugs [1]. In 2008, through the Containment Project, the World Health Organization (WHO) and its partners, with extra funding from the Bill & Melinda Gates Foundation, promoted a policy of containment and eventual elimination of artemisinin-resistant *P. falciparum* in the Thailand–Cambodia border area [2]. This strategy was subsequently expanded to target malaria elimination across the GMS for *P. falciparum* by 2025 and all malaria by 2030 [3]. The National Malaria Elimination Strategy for Thailand 2017–2026 envisions the elimination of malaria by 2024 [4, 5].

Thailand is situated between two discrete malaria transmission regions, with the north-east provinces in a zone comprising Cambodia, southern Vietnam, and southern Laos; and the western provinces in a zone with eastern Myanmar and northern Malaysia. Although *P. falciparum* and *Plasmodium vivax* are the dominant parasites, malaria cases caused by *Plasmodium malariae*, *Plasmodium ovale*, and *Plasmodium knowlesi* also occur, as do mixed infections. Malaria transmission in Thailand is concentrated in forested areas along these international borders, with forest workers, displaced people, refugees, migrants, and police and military personnel most exposed to risk [6]. High population mobility in these areas can cause malaria to re-establish in villages where the disease has been eliminated, and civil unrest in the south of the country has hampered malaria control activities [7]. Despite these challenges, malaria incidence has declined overall since 2000, though more rapidly for *P. falciparum* compared with *P. vivax* [8]*.*

Targeting malaria elimination has required repositioning of the health system in Thailand. At a national level, the Department of Disease Control is responsible for malaria policy and strategy, whereas the Division of Vector Borne Diseases (DVBD) undertakes capacity building and provides technical support, including managing the Malaria Information System (MIS)—a national database for malaria surveillance and monitoring [9]. The electronic MIS was initially designed to track artemisinin resistance while also modernizing Thailand’s existing paper-based reporting system [2]. In addition to malaria diagnosis and treatment, case investigation is conducted to determine the origin of the infection, thereby enabling identification, classification, and elimination of transmission foci [10]. In areas deemed to be at risk, active case detection is employed and vector control measures enhanced, with the aim of interrupting transmission [4]. This case-based malaria surveillance is captured in the MIS, providing near real-time information, which the DVBD uses to stratify malaria risk down to the village level. These detailed and timely data allow local authorities to understand the potential for malaria outbreaks, and to respond quickly should they occur [4].

Given the constant threat of drug-resistant *P. falciparum*, a gap in surveillance would be unacceptable. Ineffective anti-malarial therapy drives the spread of resistant parasites, so it is critical to detect changes in *P. falciparum* susceptibility to deployed artemisinin-based combinations and switch regimens promptly where necessary to maintain momentum towards malaria elimination [11]. Therapeutic efficacy studies (TES) have been the primary tool deployed to track anti-malarial efficacy and develop responses to *P. falciparum* drug resistance across the GMS. Since 2000, the DVBD monitored drug efficacy from in vivo TES conducted in Thailand with mefloquine-artesunate combination therapy for *P. falciparum* malaria [12]. The aims of the subsequent multipronged Containment Project included increasing surveillance to provide information for the containment of artemisinin-resistant parasites [2]. By 2011, the Containment Project had supported surveillance of patients who tested positive after 3 days of treatment, and it had developed systematic processes for cross-border investigation and follow-up [12]. However, the decline in malaria incidence in Thailand means that it is more difficult to recruit the minimum patient sample size for TES—in low transmission settings, at least 50 patients are required, ideally recruited within a single malaria season [13].

*Plasmodium vivax* control is complicated by the persistence of hypnozoites that remain dormant in the host’s liver before activation [14]. Radical cure consists of chloroquine to address the blood-stage infection plus primaquine to target hypnozoites. It is not operationally possible to determine whether *P. vivax* recurrences are recrudescence (chloroquine failure), relapse (primaquine failure), or re-infection. However, chloroquine should suppress asexual parasitaemia from relapse for approximately one month [15], so recurrence on or before day 28 can be considered chloroquine treatment failure. Failures after day 28 are conservatively regarded as primaquine failures but could be caused by re-infection. Follow-up of 3 months is both sufficient to assess primaquine efficacy and operationally feasible in Thailand. By preventing relapses, radical cure both reduces malaria incidence and interrupts transmission, so ensuring effective therapy is a key component of malaria elimination.

Integrated drug efficacy surveillance (iDES) is a novel approach that incorporates drug resistance monitoring as part of routine case-based surveillance and response. iDES expanded the initiatives undertaken in the Containment Project, requiring that all malaria cases, symptomatic or asymptomatic, have a laboratory confirmed malaria diagnosis and receive treatment according to national guidelines, with parasitological follow-up to ensure parasite clearance. iDES aims to support evidence-based strategic policy development and operational decision making to realize malaria elimination while ensuring patient outcomes. This study examines the trends and status of malaria in Thailand, the implementation of iDES, initial measures of programme performance, and the potential for further development. The contribution of iDES data to decision making in the context of malaria elimination is also discussed.

## Methods

### iDES aims and implementation

Thailand’s malaria elimination strategy requires that all patients have malaria diagnosed by microscopy or rapid diagnostic test, receive supervised treatment in adherence to the national treatment guidelines, and are followed up four times to ensure cure [5, 16]. iDES supports these aims by monitoring adherence to treatment guidelines, tracking follow-up rates, and recording clinical and parasitological outcomes as part of routine case management [17].

From May 2017, the iDES protocol, updated from previous in vivo studies, was piloted in three provinces in northern Thailand (Chiang Mai, Chiang Rai, and Mae Hong Son), with expansion to eight provinces in fiscal year 2017 (FY2017; i.e., 1 October 2017 to 31 September 2018) [17]. National rollout of the iDES protocol commenced in FY2018. The MIS was upgraded to allow data capture from iDES activities, and analytics and visualizations were developed to allow routine data interrogation [9]. The iDES system aims to systematically capture epidemiological and laboratory data from all patients diagnosed with malaria in Thailand as part of routine malaria care. The iDES protocol outlines aims, treatment, follow-up, data recording, and sample collection and submission [17]. The methods described here are consistent with ongoing iDES management and operational procedures.

### Treatment

All treatment was provided free of charge in all health facilities responsible for malaria case management—malaria posts, border malaria posts, malaria clinics, health-promoting hospitals, and public and private hospitals [18]. Treatment was prescribed as per the Practice Guidelines for the Treatment of Patients with Malaria, Thailand, 2019 (Table 1) [16]. Artesunate–mefloquine was replaced in 2015 with dihydroartemisinin–piperaquine as first-line therapy for *P. falciparum* malaria, with rollout in 2016. In FY2019, dihydroartemisinin–piperaquine was withdrawn from Sisaket and Ubon Ratchathani Provinces in north-east Thailand owing to the high treatment failure rates observed from iDES and confirmed by another study [19]; it was replaced with pyronaridine–artesunate. Single-dose primaquine (30 mg) was required for *P. falciparum* cases to clear gametocytes [20]. Radical cure of *P. vivax* or *P. ovale* malaria was recommended to clear hypnozoites with chloroquine plus 14-day primaquine (0.25 mg/kg/day). Note that primaquine was not given if the patient was pregnant or under 2 years of age. In the case of confirmed glucose-6-phosphate dehydrogenase (G6PD) deficiency, an 8-week treatment of weekly primaquine (0.75 mg/kg/week) was recommended for *P. vivax*/*ovale* radical cure. Any patient with a blood smear suspected to be *P. knowlesi* was referred to the district hospital for polymerase chain reaction (PCR)-based diagnosis and treatment.

**Table 1:** National treatment guidelines for malaria in Thailand

*P. falciparum*
First-line: DHA–PIP for 3 days + PQ single dose (30 mg or 0.5 mg base/kg, started on day 1 pending patient’s condition) (except for Sisaket and Ubon Ratchathani, where PY–AS for 3 days + PQ single dose was adopted in FY2019) Second-line (ACT): 1. PY–AS 3 days + PQ single dose; 2. AL for 3 days + PQ single dose; 3. AS–MQ for 3 days + PQ single dose Second-line (non-ACT): 1. Quinine + clindamycin–doxycycline–tetracycline for 7 days + PQ single dose; 2. Atovaquone–proguanil for 3 days + PQ single dose
*P. vivax* or *P. ovale*
First-line: CQ for 3 days + PQ (0.25 mg base/kg per day, started on day 3) for 14 days Second-line: DHA–PIP for 3 days + PQ for 14 days
*P. malariae* or *P. knowlesi*
First-line: CQ for 3 days Second-line: DHA–PIP for 3 days
*P. falciparum* plus *P. vivax* or *P. ovale*
DHA–PIP for 3 days + PQ for 14 days
*P. falciparum* plus *P. malariae* or *P. knowlesi*
DHA–PIP for 3 days + PQ single dose
Severe malaria
First-line: Artesunate injection within 24 h, followed by first-line/second-line regimen when tolerated + supportive care Second-line: Quinine injection within first 24 h followed by first-line/second-line regimen when tolerated + supportive care
Uncomplicated malaria in pregnancy 1st trimester^a^
Quinine + clindamycin for 7 days (*P. falciparum* or *P. knowlesi*) CQ for 3 days (*P. vivax, P. ovale*, or *P. malariae*)
Uncomplicated malaria in pregnancy 2nd or 3rd trimester^a^
DHA–PIP for 3 days (*P. falciparum* or *P. knowlesi*) CQ for 3 days (*P. vivax, P. ovale*, or *P. malariae*)

^a^Primaquine and doxycycline–tetracycline are contraindicated during pregnancy

*ACT* artemisinin-based combination therapy, *DHA–PIP* dihydroartemisinin–piperaquine, *PQ* primaquine, *PY–AS* pyronaridine–artesunate, *AS–MQ* artesunate–mefloquine, *AL* Artemether–lumefantrine, *CQ* chloroquine

### Follow-up

Patients with falciparum malaria infection were followed up at days 3, 7, 28, and 42 (from the day of diagnosis and prescribed treatment) and those with vivax malaria at days 14, 28, 60, and 90. For *P. falciparum* cases, the PCR genotyping results could be added later to the patient record (see below). For each follow-up visit, the clinical team noted the date, patient temperature, microscopy findings, and confirmation of outcome as recrudescence or re-infection. Patients were asked if they had been consuming the anti-malarial drugs as prescribed, and the drug bag was requested for observation as a proxy of treatment compliance.

### Data recording

**Fig. 1:**
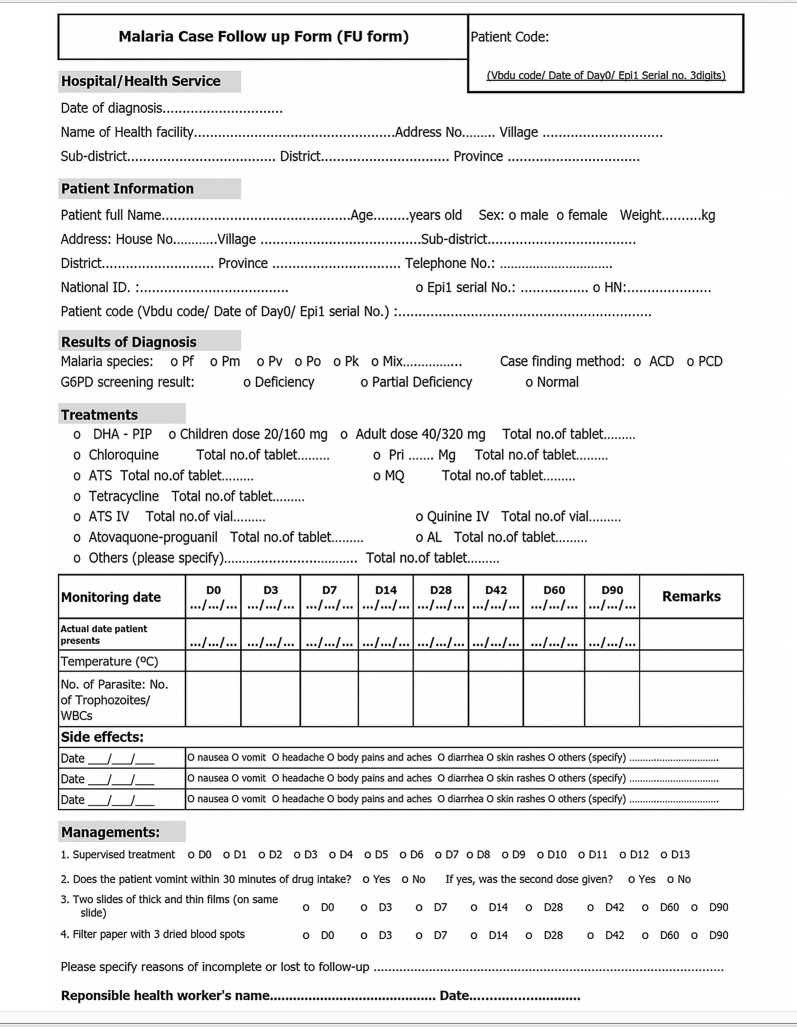
Malaria case follow-up form

The officer from the laboratory reference centre was responsible for data recording, either at a reference laboratory (DVBD) or a regional laboratory (Department of Disease Control). The reporting requirements were consistent with those used in TES to enable comparison with historical data and between countries [13]. The patient’s name, age, gender, weight, height, temperature, address, and residency background [i.e., resident Thai, long-term migrant (≥ 6 months residency), or short-term migrant (< 6 months residency)], were recorded, as well as the date that blood samples were taken and the date on which the person visited the clinic. Malaria species, parasite density, and the presence of gametocytes were assessed using standard methods by trained microscopists and reported [13, 21]. The Thailand Ministry of Public Health organizes regular quality control microscopy testing and training, including competency assessments and training of trainers, with assistance from partner agencies to develop standard operating procedures [22]. The prescribed anti-malarial drug and number of tablets/capsules were noted. All of these data were captured on the Malaria Case Follow-Up form and stored in the MIS (Fig. 1).

### Sample collection and submission

Blood spots were collected onto filter paper from all patients at first consultation and at follow-up visits. Samples were sent to regional and national laboratories as part of the microscopy quality assurance system. In the case of *P. falciparum*, PCR genotyping was used to compare samples from the initial and recurrent infection to differentiate recrudescence from re-infection, according to published methods [23]. For *P. falciparum* cases, blood samples were also collected for analysis of *kelch 13* gene mutations associated with artemisinin resistance and *Pfmdr1* gene copy number [24, 25]. Data on molecular markers will be reported separately.

### Outcomes and statistical analysis

The primary outcome of the analysis was the crude drug efficacy rate among patients who had microscopically confirmed malaria with an identified *Plasmodium* species at baseline, received first-line treatment (dihydroartemisinin–piperaquine + primaquine for *P. falciparum* or chloroquine + primaquine for *P. vivax*), and had at least one post-baseline follow-up visit*. Plasmodium falciparum* treatment failure was defined as microscopically confirmed parasite reappearance within 42 days of first-line therapy. Crude cure rates were reported, as PCR data were sparse. For *P. vivax*, confirmed parasite reappearance within 28 days was considered chloroquine treatment failure, whereas reappearance at day 60 and day 90 was conservatively considered primaquine treatment failure (though could be caused by re-infection). Drug efficacy was estimated using Kaplan–Meier methods. Patients were censored if they had a re-infection with a different species from their initial infection or at their final treatment outcome assessment. In order to assess programmatic efficacy, all re-treatments with first-line therapy following failure were considered to be new malaria episodes, and the patients were requested to report for another four follow-up visits to ensure complete cure. All other outcomes were reported using descriptive statistics. Data were downloaded from the MIS on 16 November 2020. Statistical analysis used Stata (version 16) and GraphPad Prism (version 9.0.0).

## Results

### Malaria trends

**Fig. 2:**
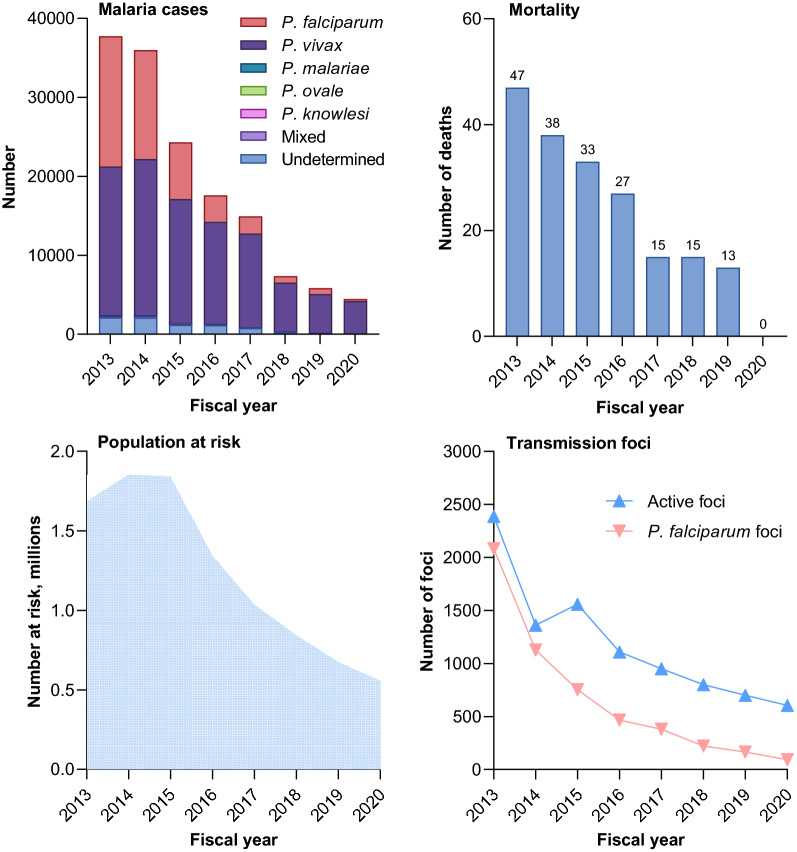
Malaria trends in Thailand FY2013–FY2020. Malaria mortality figures for FY2020 are provisional until officially approved by the Division of Planning and Strategy, Ministry of Public Health, Thailand

Between FY2013 and FY2020, there was an overall 88.1% decrease in malaria incidence from 37,741 to 4474 cases (Fig. 2). There was a sustained decline in the incidence of both *P. falciparum* and *P. vivax* malaria, but with a marked change in the relative proportions of infections caused by these two parasites; in FY2013, 43.7% (16,494/37,741) of infections were caused by *P. falciparum,* whereas in FY2020, this figure had declined to 5.7% (257/4474) (Fig. 2). Over the same period, malaria mortality declined from 47 to 0 deaths (Fig. 2). The number of active malaria foci (defined as subvillages with ongoing malaria transmission) also contracted, from 2,387 in FY2013 to 605 in FY2020, representing a 74.7% decrease (Fig. 2).

### Current malaria situation

**Fig. 3:**
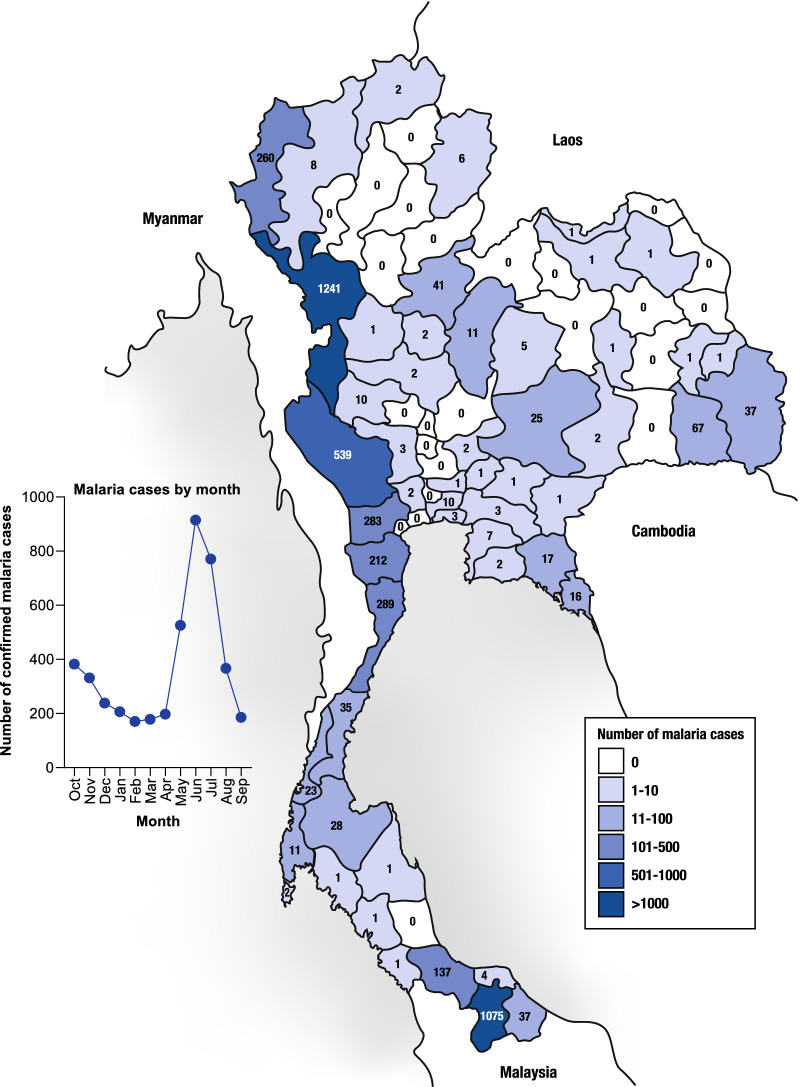
Temporal and geographical distribution of malaria cases in Thailand FY2020

From data recorded in the MIS, in FY2020 there were 4474 malaria cases, representing a case incidence rate of 0.067/1000 population, against the FY2020 target for malaria elimination of 0.22/1000 population [5]. *Plasmodium falciparum* accounted for 5.7% (257/4474) of cases and *P. vivax* for 91.6% (4099/4474) (Fig. 2). The remaining cases were classified as *P. malariae* (n = 53), *P. knowlesi* (n = 11), or mixed (n = 18) (Fig. 2). For 36 cases, the *Plasmodium* species was undetermined, and no *P. ovale* cases were recorded. Malaria incidence was concentrated along the western border that Thailand shares with Myanmar, particularly Tak and Kanchanaburi Provinces; in the south of the country bordering Malaysia in Yala Province; and in the northeast bordering Cambodia in Sisaket and Ubon Ratchathani Provinces (Fig. 3). The peak malaria transmission season occurred between April and September, with a lower peak in October (Fig. 3).

**Fig. 4:**
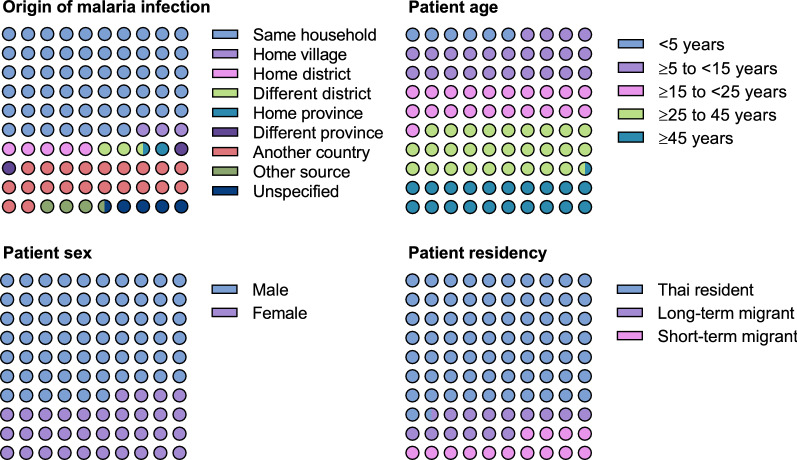
Characteristics of malaria cases in Thailand FY2020. Each square represents 100% and each dot 1% of the population. Residency background was categorized as resident Thai, long-term migrant (**≥ **6 months residency), or short-term migrant (< 6 months residency)

Most malaria infections occurred in individuals who were at least 15 years old (70.2% [3142/4474]), and around two-thirds occurred in males (65.9% [2950/4474]) (Fig. 4). Most infections were acquired in the same household (56.8% [2543/4474]), but around a fifth of cases were imported (21.3% [954/4474]) (Fig. 4). Thai residents accounted for 71.3% (3190/4474) of cases, with the remaining cases split between long-term migrants (14.9% [666/4474]) and short-term migrants (13.8% [618/4474]) (Fig. 4).

### Follow-up rates and adherence to national treatment guidelines

**Fig. 5:**
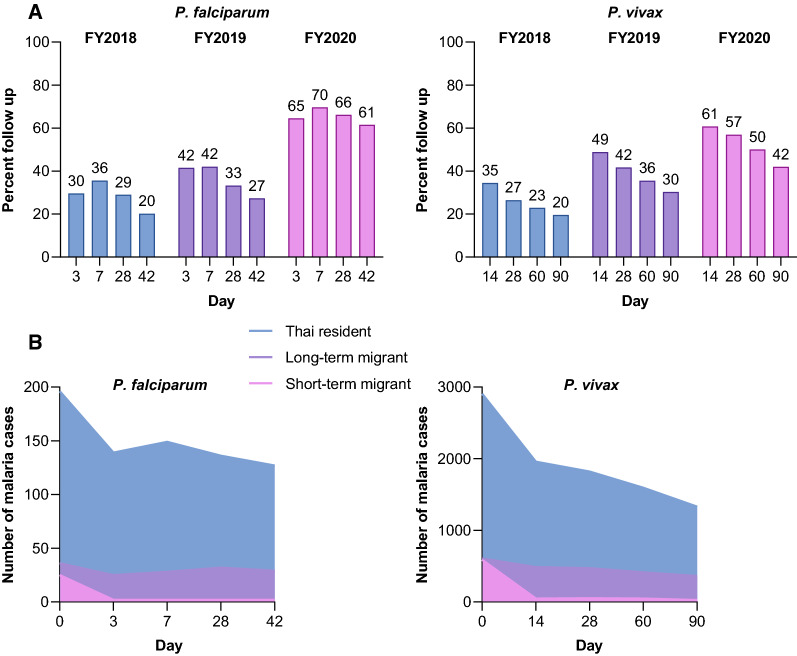
Malaria case follow-up rates in Thailand. **A** All patients FY2018–FY2020, and **B** By patient origin in FY2020

The proportion of patients with outcomes recorded at day 42 for *P. falciparum* malaria and for 28 days for *P. vivax* malaria has been increasing each year since the national launch of iDES (Fig. 5A). In FY2020, 61.5% (158/257) of *P. falciparum* cases had a treatment outcome recorded on day 42, and 57.2% (2344/4099) of *P. vivax* cases had a day 28 outcome recorded (Fig. 5A). For *P. falciparum* malaria, day 42 follow-up rates were highest for long-term migrants (80.6% [29/36]) and Thai residents (64.8% [127/196]), but lower for short-term migrants (8.0% [2/25]) (Fig. 5B). A similar pattern was observed for *P. vivax* malaria, with 77.6% (471/607) of long-term migrants, 62.7% (1821/2905) of Thai residents, and 8.9% (52/587) of short-term migrants followed up at day 28 (Fig. 5B).

The proportion of evaluable patients who received anti-malarial therapy aligned with national treatment guidelines improved over the iDES period, from 70.7% (4212/5956) in FY2018, to 77.2% (4103/5315) in FY2019, and 84.2% (3549/4215) in FY2020.

### Anti-malarial drug efficacy

Anti-malarial drug efficacy was assessed for those patients who received at least one dose of first-line treatment for *P. falciparum* (dihydroartemisinin–piperaquine + primaquine) or *P. vivax* (chloroquine + primaquine) malaria, and who had at least one follow-up visit. Recurrences occurred in different provinces in different years, though Sisaket, Tak, and Yala had *P. vivax* recurrences in all three iDES years (Table 2). *Plasmodium vivax* recurrences also varied by follow-up day, with the majority of positive tests occurring on days 60 and 90 (Table 3), suggesting either primaquine treatment failure or re-infection. In FY2020, only 3.8% (2/52) of *P. vivax* recurrences occurred on or before day 28, suggesting high efficacy for chloroquine.

**Table 2:** Summary of malaria recurrences by year, species, and province

Species and province	Recurrences, n/N (%)		
	FY2018	FY2019	FY2020
*P. falciparum* overall	13/249 (5.2)	14/379 (3.7)	3/167 (1.8)
Chanthaburi	–	–	2/6 (33.3)
Kamphaeng Phet	–	1/1 (100)	–
Phangnga	1/2 (50.0)	–	–
Sakaeo	–	1/3 (33.3)	–
Sisaket	8/59 (13.6)	10/49 (20.4)	–
Surat Thani	2/33 (6.1)	–	1/15 (6.7)
Surin	1/4 (25.0)	1/7 (14.3)	–
Tak	–	1/52 (1.9)	–
Ubon Ratchathani	1/10 (10.0)	–	–
*P. vivax* overall	66/2048 (3.2)	58/2206 (2.6)	52/2448 (2.1)
Chachoengsao	2/42 (4.8)	–	–
Chon Buri	1/6 (16.7)	–	–
Kanchanaburi	1/143 (0.7)	3/235 (1.3)	–
Mae Hong Son	3/184 (1.6)	6/199 (3.0)	–
Nakhon Ratchasima	–	2/2 (100)	1/19 (5.3)
Phetchaburi	–	2/79 (2.5)	4/124 (3.2)
Phitsanulok	–	–	2/26 (7.7)
Prachin Buri	1/22 (4.5)	–	–
Prachuap Khiri Khan	–	2/65 (3.1)	6/110 (5.5)
Ratchaburi	–	7/117 (6.0)	11/182 (6.0)
Sisaket	36/200 (18.0)	24/198 (12.1)	5/41 (12.2)
Tak	20/385 (5.2)	8/398 (2.0)	21/703 (3.0)
Yala	2/506 (0.4)	4/523 (0.8)	2/559 (0.4)

Only provinces with malaria recurrences are shown. Recurrences are shown for patients who had at least one follow-up visit for *P. falciparum* malaria up to and including day 42 and received dihydroartemisinin–piperaquine plus primaquine; and for patients who had at least one follow-up visit for *P. vivax* malaria up to and including day 90 and received chloroquine plus primaquine

**Table 3:** Summary of *P. vivax* malaria recurrences by year and follow-up day

Species and follow-up day	Recurrences, n/N (%)		
	FY2018	FY2019	FY2020
*P. vivax* overall	66/2048 (3.2)	58/2206 (2.6)	52/2448 (2.1)
Day 14	1 (1.5)	7 (12.1)	2 (3.8)
Day 21	2 (3.0)	1 (1.7)	0
Day 28	20 (30.3)	10 (17.2)	0
Day 35	0	1 (1.7)	0
Day 42	0	0	1 (1.9)
Day 60	36 (54.5)	31 (53.4)	21 (40.4)
Day 90	7 (10.6)	8 (13.8)	28 (53.8)

Recurrences are shown for patients with at least one follow-up visit for *P. vivax* up to and including day 90 who received chloroquine plus primaquine

**Fig. 6:**
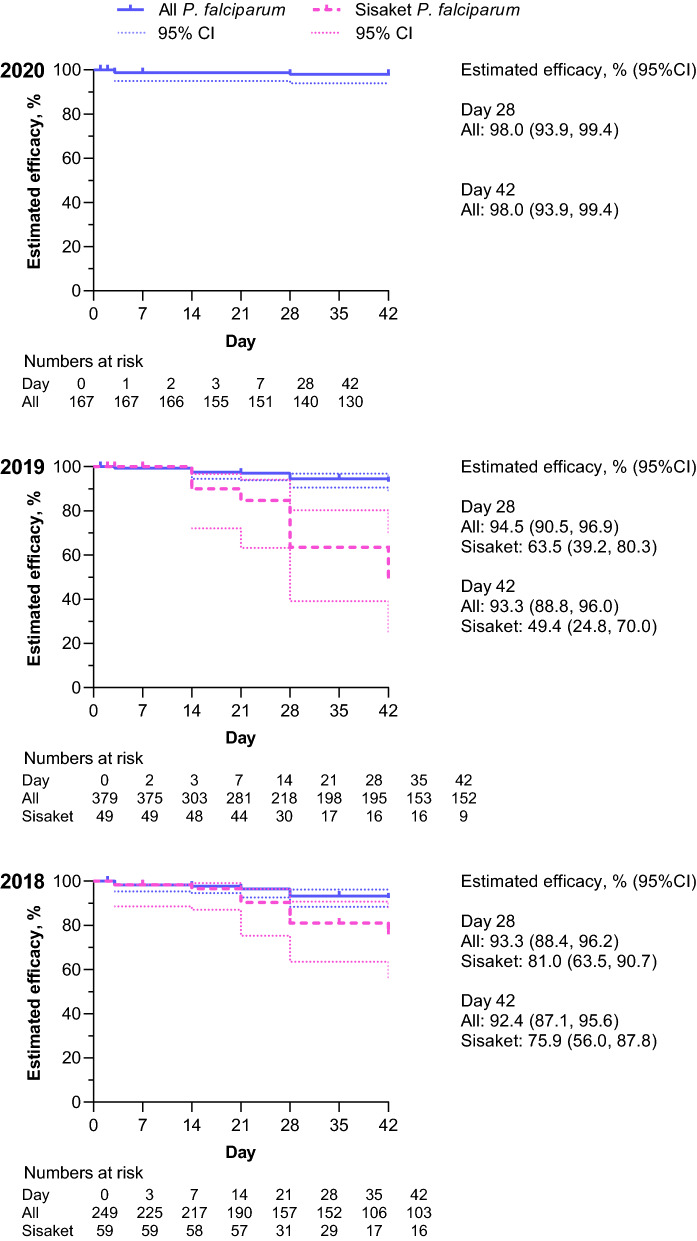
Efficacy of dihydroartemisinin–piperaquine plus primaquine against *P. falciparum* malaria in Thailand FY2018–FY2020. Data are Kaplan–Meier estimates for patients who had *P. falciparum* monoinfection, received at least one dose of both dihydroartemisinin–piperaquine and primaquine, and attended at least one follow-up visit. In Sisaket Province, pyronaridine–artesunate was rolled out as a new first-line treatment in FY2020, so data for this province are not shown for that year (see text)

For *P. falciparum* malaria, day 42 efficacy (Kaplan–Meier) was 92.4% (95%CI 87.1, 95.6) in FY2018, 93.3% (95%CI 88.8, 96.0) in FY2019, and 98.0% (95%CI 93.9, 99.4) in FY2020 (Fig. 6). Most falciparum recurrences occurred in Sisaket Province, with day 42 efficacy rates of 75.9% (95%CI 56.0, 87.8) in FY2018 and 49.4% (95%CI 24.8, 70.0) in FY2019 (Fig. 6). In FY2018, all eight treatment failures in Sisaket occurred in different individuals. In FY2019, ten treatment failures occurred in six patients, three of whom received repeated treatment with dihydroartemisinin–piperaquine rather than second-line therapy. However, even when only the first treatment episode was considered for these patients, the day 42 efficacy rate in Sisaket in FY2019 was 61.5% (95%CI 31.2, 81.7). Based on the unacceptably high clinical failure rate in Sisaket, in February 2019 pyronaridine–artesunate replaced dihydroartemisinin–piperaquine as first-line therapy against uncomplicated falciparum malaria in Sisaket and neighboring Ubon Ratchathani, with rollout in FY2020. In FY2020, there were only three *P. falciparum* cases in Sisaket and Ubon Ratchathani. Two cases received pyronaridine–artesunate, with no recurrence. One received dihydroartemisinin–piperaquine; after parasite reappearance at day 60, the case was successfully treated with pyronaridine–artesunate.

**Fig. 7:**
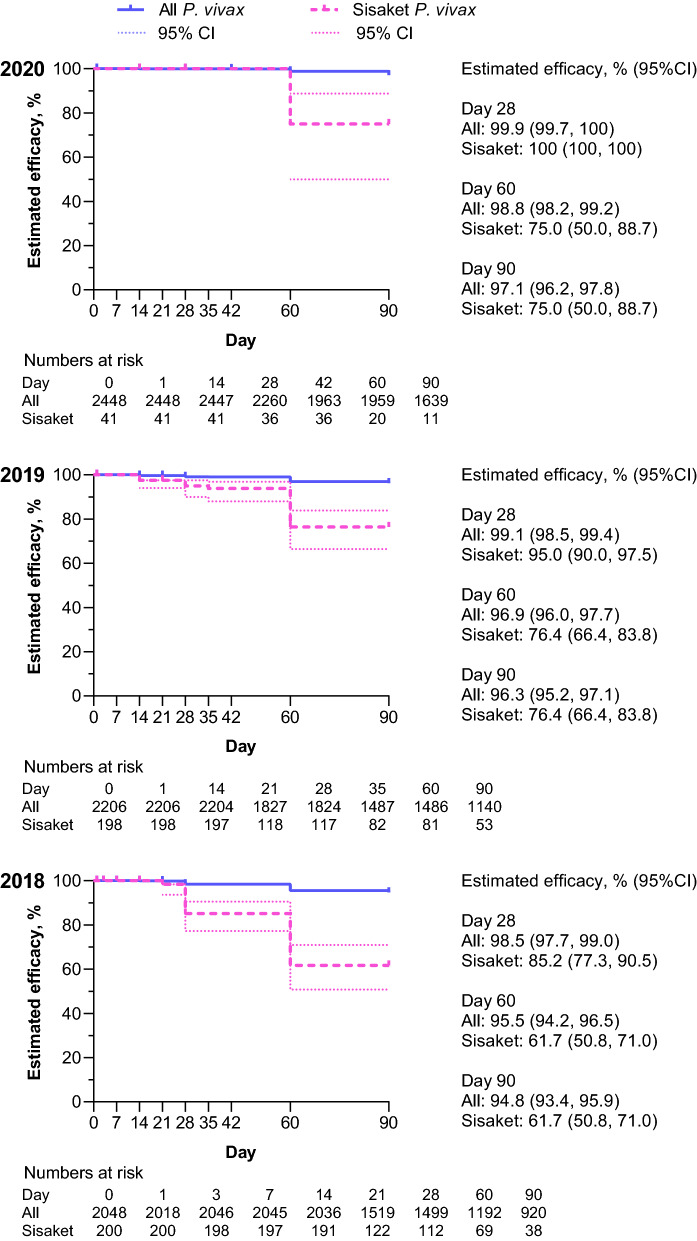
Efficacy of chloroquine plus primaquine for *P. vivax* malaria in Thailand FY2018–FY2020. Data are Kaplan–Meier estimates for patients who had *P. vivax* monoinfection, received at least one dose of both chloroquine and primaquine, and attended at least one follow-up visit. Recurrences occurring on or before day 28 were considered chloroquine treatment failures. Recurrences occurring after day 28 until day 90 were assumed to be primaquine treatment failures as a conservative analysis but could be caused by re-infection

For *P. vivax* malaria, chloroquine/primaquine day 28 efficacy was at least 98% across all iDES years, suggesting good clinical efficacy for chloroquine (Fig. 7). Day 60 efficacy was > 95%, and Day 90 efficacy was 94.8% (95%CI 93.4, 95.9) in FY2018, but improved to 97.1% (96.2, 88.7) in 2020 which suggests good overall primaquine efficacy (Fig. 7). Sisaket Province proportionally had the greatest effect on the overall failure rate. Day 28 efficacy in Sisaket was 85.2% (95%CI 77.3, 90.5) in FY2018, but improved to 95.0% (95%CI 90.0, 97.5) in FY2019, and 100% in FY2020 (Fig. 7). However, day 60 and day 90 efficacy in Sisaket remained sub-optimal, with both at 61.7% (95%CI 50.8, 71.0) in FY2018, 76.4% (95%CI 66.4, 83.8) in FY2019, and 75.0% (95%CI 50.0, 88.7) in FY2020 (Fig. 7).

## Discussion

Thailand has made significant progress towards malaria elimination, surpassing its 2020 milestone reductions for both malaria incidence and mortality [8]. However, many areas remain receptive to malaria, and re-establishment of transmission continues to be a significant risk across most of the country [26].

It was clear that as malaria incidence declined in Thailand, TES would no longer be feasible in many provinces. The reorganization of the health system to target malaria elimination offered an opportunity to integrate drug efficacy surveillance. However, retroactive adaptation of the MIS to incorporate the iDES module was complex and has required continued refinement of the platform to adapt to changing epidemiology and corresponding data needs. The DVBD can examine the data in almost real time, thereby enabling limited resources to be targeted to the provinces, villages, or health facilities most in need of support to improve iDES compliance. The DVBD and its partners are working to further improve data visualizations to facilitate the data analyses most needed by subnational officers, such as health care workers interested in patient follow-up and treatment outcomes.

In order to move from TES to iDES, it was necessary for Thailand to have a strong case-based surveillance system and the capacity for universal and supervised anti-malarial treatment. For countries that have not yet reached the elimination phase, TES remains the most appropriate drug efficacy surveillance method [18]. For iDES to have an impact on policy, other aspects of the health system need to be aligned for delivering different anti-malarial drugs to different regions. For example, purchasing and supply logistics, laboratory capacity and coordination, health financing, communication with prescribers, community outreach, and patient education. Thus, iDES cannot just be appended to an information system, but instead must be fully integrated into all health system processes.

Rapid diagnostic tests for malaria have been an invaluable tool in areas of high-to-moderate transmission to identify cases and direct appropriate therapy. However, iDES requires microscopic malaria diagnosis to confirm parasite clearance. Maintaining microscopy skills in an elimination setting is challenging as some laboratories see very few cases. With sustained support from external funding partners, Thailand has invested in a strong cadre of trained microscopists stationed throughout the country, and 33 professionals hold current expert certification from the WHO. However, other countries in the GMS and elsewhere may need to consider how to build these skills as malaria burden reduces, and as an iDES system becomes the recommended programme for case-based surveillance and follow-up.

Despite being included in the iDES protocol, the collection of dried blood spots for PCR analysis of *P. falciparum* has been sporadic, with difficulties in managing their storage and processing. Thus, only crude efficacy rates are reported here, but in a country aiming for elimination it is expected that all recurrences are recrudescences. The collection of samples for molecular resistance markers has also been sub-optimal while subnational officers gain new skills. The DVBD anticipates enhanced molecular surveillance and streamlined processes to triangulate clinical and laboratory data as a National Reference Laboratory database is developed and with additional training for health workers and laboratory staff.

Follow-up rates for iDES have been increasing steadily since its introduction, supported by a network of village health volunteers and through community education. High burden provinces in western Thailand (Fig. 3) have lower follow-up rates, as does the crucial Sisaket Province. Also, follow-up rates among short-term migrants have been consistently low. Maintaining contact with this population is challenging, and cross-border collaboration between countries may be required to ensure patient outcomes. There is the potential to incorporate mobile health data (mHealth) within iDES [27], boosting follow-up rates by expanding coverage of malaria follow-up services to the household and individual levels via patients’ mobile devices. iDES could also be enhanced by complementary research to identify potential bottlenecks to patient follow-up—such as patient resistance or forgetfulness, provider lack of knowledge, poor adherence, or issues with recording and reporting. A positive sign is that in FY2020, malaria case follow-up and iDES showed resilience to disruption caused by the novel coronavirus disease (COVID-19) epidemic, given that both follow-up rates and data capture improved.

Adherence to the national treatment guidelines is necessary to optimize patient outcomes and to support malaria elimination. Although adherence rates have been improving, further progress will require additional resources. For example, Thailand’s village health volunteers are key in accessing remote locations and engaging with patients on a personal level, and additional training of these health workers is planned in FY2021, alongside iDES capacity building. In Sisaket, iDES identified issues related to re-treatment with first-line therapy following failure, leading to subsequent treatment failure. These repeated treatment failures are programmatic and may result from stock-outs of second-line anti-malarial therapies, patients presenting at different clinics, or patient or prescriber choice. Being able to find and interrogate these cases allows interventions to be targeted at the causes of non-adherence to treatment guidelines in specific locations.

Although follow-up rates and treatment adherence are not perfect in terms of ensuring individual case outcome, data penetration has been sufficient to enable policy decisions on anti-malarial drug treatment at the provincial level, with dihydroartemisinin–piperaquine switched to pyronaridine–artesunate for two provinces. Although pyronaridine–artesunate shows high efficacy in regions in the GMS where multidrug-resistant *P. falciparum* parasites are prevalent [28–31], normally a TES study would be conducted to support a drug treatment policy change. Instead, pyronaridine–artesunate efficacy in Sisaket and Ubon Ratchathani will be monitored in FY2021 through ‘intensified iDES’ (Box 1), which aims to optimize data gathering, given the anticipated low number of *P. falciparum* cases.

**Box 1:** Intensified iDES to support treatment policy change

100% adherence to the national treatment guidelines
No stock-outs of drugs
Additional training for treatment providers to ensure daily supervised drug intake
Target to achieve > 90% of follow-up days
iDES standard operating procedures are followed with increased frequency of monitoring
Adequate patient support for follow-up visits (providing transport, etc.)
Quality control on all microscopy slides
Collection of all day 0 dried blood spots and at recurrence for PCR and molecular markers
Follow-up of all treatment failures
Integration of laboratory data and the results of molecular markers to the online system

iDES data also underline the importance of *P. vivax* malaria elimination. Although generally high efficacy rates were observed, a disparity was evident in Sisaket versus other provinces. The high day 28 failure rate in Sisaket in FY2018 suggests sub-optimal chloroquine efficacy, though efficacy was 100% in FY2020. Mutations associated with chloroquine resistance have been detected in *P. vivax* isolates from Thailand [32]. Notably, Sisaket borders Cambodia, where chloroquine was abandoned in 2012 because of parasite resistance, being replaced first by dihydroartemisinin–piperaquine, and then by mefloquine-artesunate in 2017. There is some evidence of increasing chloroquine susceptibility in Cambodian clinical isolates [33], which could explain the improved day 28 efficacy observed in Sisaket between FY2018 and FY2020. However, day 90 efficacy has remained unacceptably low in the province, suggesting sub-optimal primaquine efficacy. Although primaquine treatment should be fully supervised, drug consumption cannot be verified and poor primaquine adherence cannot be excluded as a cause. Cytochrome 2D6 polymorphisms can affect primaquine efficacy [34], but this was not investigated. There may also be social factors in this particular region that predispose the population to *P. vivax* re-infection, despite declining case numbers. Investigations and discussions on the appropriate response to *P. vivax* malaria in Sisaket are ongoing in FY2021, and the findings may also affect the management of *P. vivax* malaria elsewhere in Thailand.

iDES has provided operationally relevant data on drug efficacy; however, there are some limitations. Most importantly, it is difficult to obtain a complete dataset for data collected routinely, which may introduce a bias towards patients that are more easily reached. As follow-up improves, this bias should diminish. Similarly, although health workers attempt to verify drug adherence, not all treatment is directly observed. Thus, although malaria recurrence in this report is attributed to treatment failure, it could be a result of poor adherence. From an operational perspective, it is valuable to be able to differentiate these two sources of recurrence, so health workers are receiving continued training to observe the drug pack as a proxy for consumption. A final consideration is that MIS data can be adjusted to reflect new information received or to correct records, and so should be regarded as cross-sectional.

Given the low and declining malaria incidence in Thailand, without iDES it is unlikely that a pattern of treatment failure would be detected in time to avert an outbreak of drug-resistant *P. falciparum*. There is evidence that iDES can support appropriate management of imported malaria as part of a comprehensive prevention of reintroduction program [35]. Maintaining iDES may be crucial for Thailand as neighbouring countries in the GMS strive for elimination in the coming years.

## Conclusion

The investment in malaria elimination in Thailand is considerable, but the potential benefits are even greater [36]. As countries approach elimination, it is likely that the most resistant parasites will be those that remain in circulation [11]. Ineffectual therapy promotes the spread of drug-resistant parasites, and risks malaria resurgence and the re-establishment of transmission. Thus, as it becomes more difficult to conduct drug resistance surveillance activities, it also becomes increasingly important to understand which drugs retain efficacy and whether treatment policies should be amended. iDES is designed to encompass all malaria cases, whether imported or indigenous; to encourage treatment compliance; and to follow patients until they are clinically cured and confirmed as parasite free. Thailand’s experience with iDES offers a pragmatic model for malaria-eliminating countries where TES is no longer feasible. iDES can be a useful approach to target malaria elimination by ensuring that all malaria patients receive appropriate treatment and are ultimately cured of malaria.

## Data Availability

All relevant data are provided in the manuscript or are available from the website of the Department of Disease Control, Ministry of Public Health Thailand: https//malaria.ddc.moph.go.th.

## Abbreviations

ACT: Artemisinin-based combination therapy
AL: Artemether–lumefantrine
AS–MQ: Artesunate–mefloquine
COVID-19: Coronavirus disease of 2019
CQ: Chloroquine
DHA–PIP: Dihydroartemisinin–piperaquine
DVBD: Division of Vector Borne Diseases
FY: Fiscal year
G6PD: Glucose-6-phosphate dehydrogenase
GMS: Greater Mekong Subregion
iDES: Integrated drug efficacy surveillance
MIS: Malaria Information System
PCR: Polymerase chain reaction
PQ: Primaquine
PY–AS: Pyronaridine–artesunate
TES: Therapeutic efficacy studies
USAID: United States Agency for International Development
WHO: World Health Organization
